# Integrative multi-omics reveals the metabolic and functional diversity of microbiomes in the gut microenvironment

**DOI:** 10.3389/fmicb.2023.1168239

**Published:** 2023-06-22

**Authors:** Shi Qiu, Zhibo Wang, Qiang Yang, Ying Cai, Yiqiang Xie, Songqi Tang, Aihua Zhang

**Affiliations:** ^1^International Advanced Functional Omics Platform, Scientific Experiment Center, International Joint Research Center on Traditional Chinese and Modern Medicine, Key Laboratory of Tropical Cardiovascular Diseases Research of Hainan Province, Hainan General Hospital, College of Chinese Medicine, Hainan Medical University, Haikou, China; ^2^Graduate School, Heilongjiang University of Chinese Medicine, Harbin, China

**Keywords:** microbiome, gut microenvironment, multi-omics, metabolic diversity, metabolite

Multi-omics profiling has been used to reveal molecular signals representing the metabolic and functional diversity of microbiomes in the gut microenvironment (Park et al., 2022; Vatanen et al., 2022a,b; Kindschuh et al., 2023). These studies have shown the correlation between gut microbiota and metabolites produced or modified by microbiomes and clarified the function of the integrative multi-omics approach in understanding the gut microbiome and its impact on human health and disease. The gut microbiota is critical for maintaining host metabolic homeostasis by regulating multiple functions. The composition and metabolism of the microbiota may play a key role in gut homeostasis. Gut microflora could produce a variety of metabolites that have a significant effect on intestinal motility and secretion (Dohnalová et al., 2022). Microbiomes have produced or modified metabolites that have become important factors with potential effects on the host (Qiu et al., 2023). Given the critical role of the gut microbiome in health and disease, it is necessary to understand the factors that influence both the microbiome and the microenvironment. The widespread influence of the functional metabolite diversity of the gut microbiome and microenvironment remains largely unknown. Multi-omics is an effective approach to evaluating the functional metabolic diversity of the gut microbiome and exploring the relationship between the intestinal flora and the host (Figure 1).

**Figure 1:**
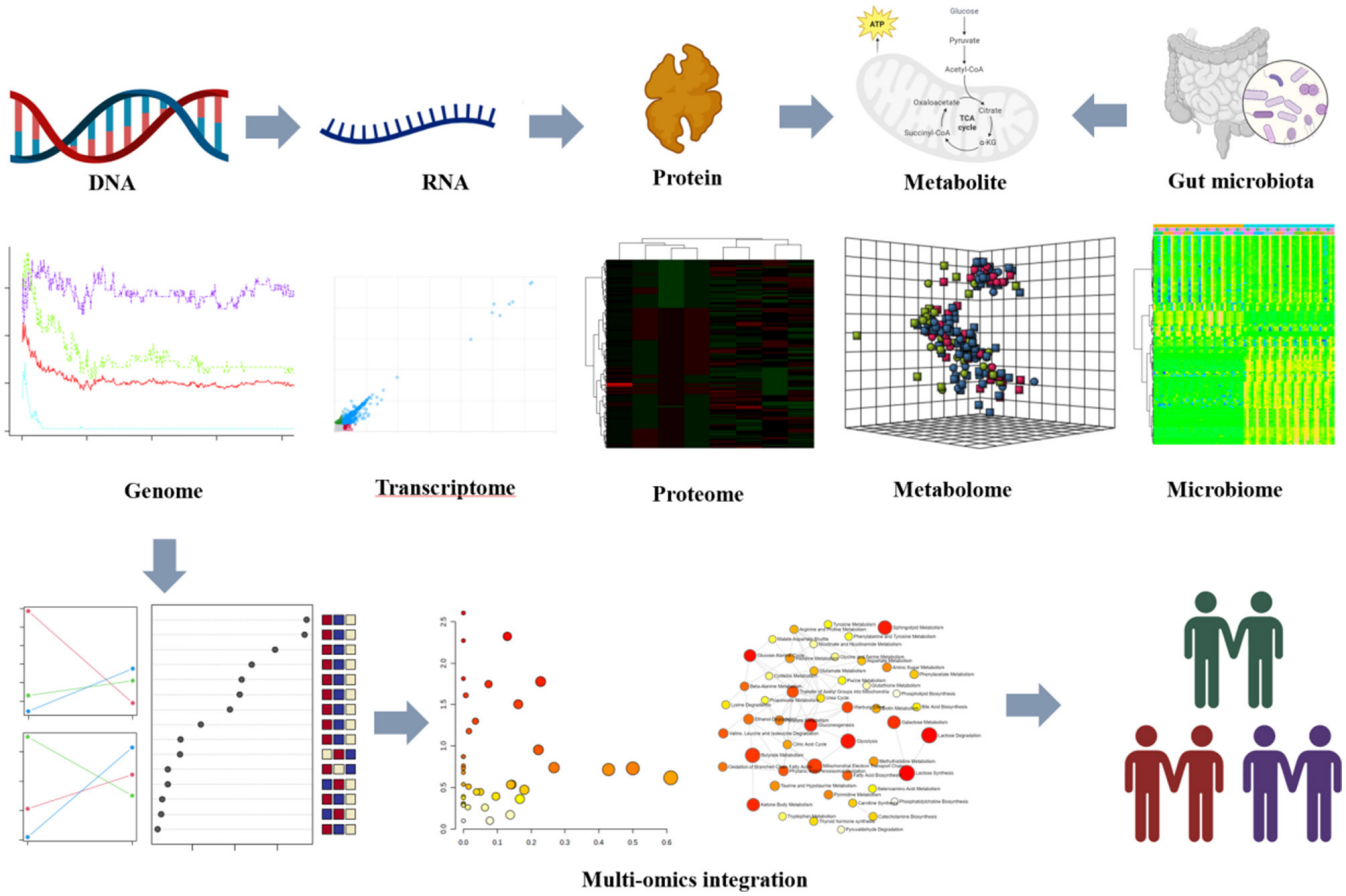
*In-vivo* multi-omics profiles reveal the metabolic and functional diversity of the gut microbiome in this microenvironment. Multi-omics platforms decoding molecular signatures of the gut microbiome were collected from patients and integrated to identify integrative functional changes. All images were obtained using the sample data provided by MetaboAnalyst 5.0, and plots were created using BioRender.

A recent study by Vatanen et al. investigated the maternal microbiome shaping the gut microbial metabolism, consisting of mother-infant samples at different stages of infancy and late pregnancy (Vatanen et al., 2022a). They examined more than 2,500 specific metabolic characteristics of the infants and found many specific associations between fecal metabolites and bacterial species. They also discovered that increases in steroid compounds and intermediates of bile acid biosynthesis are linked to pregnancy. Remarkably, in addition to the classical vertical transmission, the maternal microbiota can also shape the infant's intestinal microbiota through horizontal gene transfer events. Maternal species can influence the microenvironment of the infant microbiota through mobile genetic elements. In addition, rapid identification of microbial metabolites in infant intestines may provide an approach for further research into such microorganisms to develop targeted interventions for optimal opportunities in infancy. Future studies should further investigate the association between bacteria and metabolism and explore the specific metabolic output characteristics of bacteria. Vatanen et al. characterized the gut microbial diversity and the downstream contributions to gut metabolism by analyzing longitudinal multi-omics data from children's fecal samples (Vatanen et al., 2022b). Notably, they found that *bifidobacteria* play a leading role early in life, followed by abundant *Prevotella*. Moreover, to better understand the diversity of metabolic activities of *B. longum* clades, they also found that *B. longum* clades and numerous secondary metabolites were associated with diarrhea and early growth in children, while transitional *B. longum* was correlated with the metabolites derived from microorganisms, including citrulline, imidazole propionic acid, N-acetylputrescine, and 5-hydroxyectoine. This work provided evidence for the importance of *B. longum* subsp. Infantis and its associated metabolites in the host-microbe symbiosis and the development of the infant.

Microbial communities can produce various metabolic products to regulate host physiology and disease, but their mechanisms of action are not fully understood. In a recent study in *Cell*, Park et al. reported that butyrate derived from microorganisms is an environment-dependent inhibitor of the *Bacteroidales* and revealed its key role in the gut microbiota microenvironment (Park et al., 2022). Employing multi-omics profiles, they proved that the fitness of gut *Bacteroides* is linked to, but not independent of, the expression of acyl-CoA transferase and butyrate. It is noteworthy that the inhibitory mechanism of butyrate is related to variations in the metabolic function and regulation of acyl-CoA. Park and colleagues determined that butyrate, a specific microbial metabolite, has an inhibitory effect on *Bacteroides* and then demonstrated that acyl-CoA metabolism plays a role in metabolic defense. By identifying the specific inhibitory function of butyrate, this work proposed a relationship between growth inhibition and metabolism. The active metabolites produced by the gut microbiota and their interactions with surrounding environmental signaling molecules play a major role to regulating human health in various ways, affecting genetic, biochemical, and biological functions to maintain host metabolic homeostasis. The application of gut microbiota in regulating health benefits through metabolomics is gradually expanding. Based on second-trimester vaginal and pregnancy samples, Kindschuh et al. found multiple associations between metabolome and microbiome, including the association between bacteria and metabolites enriched in term pregnancies (Kindschuh et al., 2023). They then studied the relationship between spontaneous preterm birth (sPTB) and specific metabolites and found that four metabolites, including ethyl β-glucopyranoside, tartrate, diethanolamine, and choline, were significantly correlated with sPTB. This demonstrated the potential of vaginal metabolites as early biomarkers of sPTB. Before clinical application, the new model requires to be strengthened and further validated.

Taken together, these multi-omics studies provide excellent examples of revealing the functional metabolic diversity of microbiomes by generating cross-talk between microbiome and host. They also emphasize the effects of gut microbiota and small-molecule metabolites on pathophysiological roles related to health and disease. The gut microbiota can be considered the largest immune organ and is highly associated with systemic metabolic disorders, so targeting the microbiota may be a potential therapy for metabolic diseases. Nevertheless, these studies have paid less attention to diet and lifestyle changes that may have affected the microbiome and metabolism. Further research is needed to explore the complex microorganism-host interactions that may affect the occurrence and development of disease. Additionally, the molecular mechanisms of the metabolic functional diversity of microbiomes in the gut microenvironment remain to be determined. The upcoming expansion of the endogenous metabolite database will provide analytical opportunities to discover the relationship between microbial metabolites and the host. Artificial intelligence, machine learning, multidimensional statistical methods, and accessible analytical tools for discovering the association of microbial metabolites and metabolic functional diversity are crucial for analyzing the large amount of data generated by gut microflora and microbiome research in the gut microenvironment.

## Author contributions

SQ, ZW, QY, and YC participated in the study design, contributed to method development, and wrote the manuscript. YX, ST, and AZ provided advice on method development and analysis and contributed to drafting the manuscript. All authors contributed to the article and approved the submitted version.
